# Traveling wave solutions of the Boussinesq equation via the new approach of generalized (*G′/G*)-expansion method

**DOI:** 10.1186/2193-1801-3-43

**Published:** 2014-01-23

**Authors:** Md Nur Alam, M Ali Akbar, Harun-Or- Roshid

**Affiliations:** Department of Mathematics, Pabna University of Science and Technology, Pabna, Bangladesh; Department of Applied Mathematics, University of Rajshahi, Rajshahi, Bangladesh

**Keywords:** New approach of generalized (*G′/G*)**-**expansion method, The Boussinesq equation, Homogeneous balance, Traveling wave solutions, Nonlinear evolution equation

## Abstract

**Abstract:**

Exact solutions of nonlinear evolution equations (NLEEs) play a vital role to reveal the internal mechanism of complex physical phenomena. In this work, the exact traveling wave solutions of the Boussinesq equation is studied by using the new generalized (*G′/G*)-expansion method. Abundant traveling wave solutions with arbitrary parameters are successfully obtained by this method and the wave solutions are expressed in terms of the hyperbolic, trigonometric, and rational functions. It is shown that the new approach of generalized (*G′/G*)-expansion method is a powerful and concise mathematical tool for solving nonlinear partial differential equations in mathematical physics and engineering.

**PACS:**

05.45.Yv, 02.30.Jr, 02.30.Ik

## Introduction

Large varieties of physical, chemical, and biological phenomena are governed by nonlinear partial differential equations. One of the most exciting advances of nonlinear science and theoretical physics has been the development of methods to look for exact solutions of nonlinear partial differential equations. Exact solutions to nonlinear partial differential equations play an important role in nonlinear science, especially in nonlinear physical science since they can provide much physical information and more insight into the physical aspects of the problem and thus lead to further applications. Nonlinear wave phenomena of dispersion, dissipation, diffusion, reaction and convection are very important in nonlinear wave equations. In recent years, quite a few methods for obtaining explicit traveling and solitary wave solutions of nonlinear evolution equations have been proposed. A variety of powerful methods, such as, the inverse scattering transform method (Ablowitz and Clarkson 1991), the homogeneous balance method (Fan 2000a), the Exp-function method (He and Wu 2006; Akbar and Ali 2012), the modified simple equation method (Jawad et al. 2010; Khan et al. 2013), the novel (*G′/G*)-expansion method (Alam et al. 2014; Alam and Akbar 2014), the improved (*G′/G*)**-**expansion method (Zhang et al. 2010), the (*G′/G*)**-**expansion method (Wang et al. 2008; Bekir 2008; Zayed 2009; Zhang et al. 2008; Akbar et al. 2012), the tanh-function method (Wazwaz 2005), the extended tanh-function method (Fan 2000b; El-Wakil and Abdou 2007), the sine-cosine method (Wazwaz 2004), the modified Exp-function method (Usman et al. 2013), the generalized Riccati equation method (Yan and Zhang 2001), the Jacobi elliptic function expansion method (Liu 2005; Chen and Wang 2005), the Hirota’s bilinear method (Wazwaz 2012), the Miura transformation method (Bock and Kruskal 1979), the new generalized (*G′/G*)-expansion method (Naher and Abdullah 2013; Alam et al. 2013a; Alam and Akbar 2013a; Alam and Akbar 2013b; Alam et al. 2013b), the Cole-Hopf transformation method (Salas and Gomez 2010), the Adomian decomposition method (Adomain 1994; Wazwaz 2002), the ansatz method (Hu 2001a; Hu 2001b), the exp(–Ф(*η*))-expansion method (Khan and Akbar 2013), the method of bifurcation of planar dynamical systems (Li and Liu 2000; Liu and Qian 2001), and so on.

The objective of this article is to apply the new generalized (*G'/G*) expansion method to construct the exact traveling wave solutions of the Boussinesq equation.

The outline of this paper is organized as follows: In Section Description of the new generalized (*G'/G*)-expansion method, we give the description of the new generalized *(G'/G)* expansion method. In Section Application of the method, we apply this method to the Boussinesq equation, results and discussions and graphical representation of solutions. Conclusions are given in the last section.

## Description of the new generalized (*G′/G*)-expansion method

Let us consider a general nonlinear PDE in the form1

where *v=v*(*x,t*) is an unknown function, Ф is a polynomial in *v*(*x,t*) and its derivatives in which highest order derivatives and nonlinear terms are involved and the subscripts stand for the partial derivatives.

**Step 1:** We combine the real variables *x* and *t* by a complex variable *η*2

where *V* is the speed of the traveling wave. The traveling wave transformation (2) converts Eq. (1) into an ordinary differential equation (ODE) for *v=v*(*η*):3

where *ψ* is a polynomial of *v* and its derivatives and the superscripts indicate the ordinary derivatives with respect to η.

**Step 2**: According to possibility, Eq. (3) can be integrated term by term one or more times, yields constant(s) of integration. The integral constant may be zero, for simplicity.

**Step 3.** Suppose the traveling wave solution of Eq. (3) can be expressed as follows:4

where either *α*_*N*_ or *β*_*N*_ may be zero, but could be zero simultaneously, *α*_*i*_ (*i=*0,1,2…,*N*) and *β*_*i*_ (*i=*1,2,…,*N*) and *d* are arbitrary constants to be determined and *M*(*η*) is5

where *G=G*(*η*) satisfies the following auxiliary nonlinear ordinary differential equation:6

where the prime stands for derivative with respect to *η*; *A*, *B*, *C* and *E* are real parameters.

**Step 4**: To determine the positive integer *N*, taking the homogeneous balance between the highest order nonlinear terms and the derivatives of the highest order appearing in Eq. (3).

**Step 5:** Substitute Eq. (4) and Eq. (6) including Eq. (5) into Eq. (3) with the value of *N* obtained in Step 4, we obtain polynomials in (*d*+*M*)^*N*^ (*N*=0,1,2,…) and (*d*+*M*)^-N^ (*N*=0,1,2,…). Subsequently, we collect each coefficient of the resulted polynomials to zero, yields a set of algebraic equations for α_*i*_ (*i*=0,1,2,…,*N*) and *β*_*i*_ (*i*=1,2,…,*N*), *d* and *V*.

**Step 6**: Suppose that the value of the constants α_*i*_ (*i*=0,1,2,…,*N*), *β*_*i*_ (*i*=1,2,…,*N*), *d* and *V* can be found by solving the algebraic equations obtained in Step 5. Since the general solutions of Eq. (6) are known to us, inserting the values of α_*i*_ (*i*=0,1,2,…,*N*), *β*_*i*_ (*i*=1,2,…,*N*), *d* and *V* into Eq. (4), we obtain more general type and new exact traveling wave solutions of the nonlinear partial differential Equation (1).

**Step 7:** Using the general solution of Eq. (6), we have the following solutions of Eq. (5):

**Family 1:** When *B* ≠ 0, *ω*=*A-C* and Ω =*B*^*2*^ + 4*E*(*A-C*) > 0,7

**Family 2:** When *B* ≠ 0, *ω*=*A-C* and Ω =*B*^*2*^ + 4*E*(*A-C*) < 0,8

**Family 3:** When *B* ≠ 0, *ω* = *A-C* and Ω = *B*^*2*^ + 4 *E*(*A-C*) = 0,9

**Family 4:** When *B* = 0, *ω* = *A-C* and Δ=*ωE>0,*10

**Family 5:** When *B* = 0, *ω = A-C* and Δ = *ωE < 0,*11

## Application of the method

In this section, we will put forth the new generalized (*G'/G*) expansion method to construct many new and more general traveling wave solutions of the Boussinesq equation. Let us consider the Boussinesq equation,12

Now, we will use the traveling wave transformation Eq. (2) into the Eq. (12), which yields:13

Eq. (13) is integrable, therefore, integrating with respect to η once yields:14

where *K* is an integration constant which is to be determined.

Taking the homogeneous balance between highest order nonlinear term *v*^2^ and linear term of the highest order *v*″ in Eq. (14), we obtain *N*=2. Therefore, the solution of Eq. (14) is of the form:15

where *α*_0_, *α*_1_, α_2_, *β*_1_, *β*_2_ and *d* are constants to be determined.

Substituting Eq. (15) together with Eqs. (5) and (6) into Eq. (14), the left-hand side is converted into polynomials in (*d*+*M*)^*N*^ (N=0,1,2,.......) and (*d*+*M*)^*-N*^ (*N*=1,2,…). We collect each coefficient of these resulted polynomials to zero yields a set of simultaneous algebraic equations (for simplicity, the equations are not presented) for *α*_0_, *α*_*1*_, *α*_*2*_, *β*_1_, *β*_2_*d*, *K* and *V*. Solving these algebraic equations with the help of computer algebra, we obtain following:

**Set 1**:16

where *n*_1_ = ( - *A*^2^ + *V*^2^*A*^2^ - 12*d*^2^*ω*^2^ + 8*Eω* - 12*Bdω* - *B*^2^), *n*_2_ = ( - 2*Edω* + 3*Bd*^2^*ω* + 2*d*^3^*ω*^2^ - *EB* + *B*^2^*d*), *n*_3_ = - ( - 2*Ed*^2^*ω* + *d*^4^*ω*^2^ + 2*Bd*^3^*ω* + *E*^2^ + *B*^2^*d*^2^ - 2*BdE*), *n*_4_ = - ( - 8*EB*^2^*ω* + *V*^4^*A*^4^ - 2*V*^2^*A*^4^ - 16*E*^2^*ω*^2^ + *A*^4^ - *B*^4^), *ω* = *A* - *C*, *V*, *d*, *A*, *B*, *C*, *E* are free parameters.

**Set 2**: 17

 Where *ω = A-C, V*, *d, A, B, C, E* are free parameters.

**Set 3**: , *V*=*V*, , , α_1_=0,18

where *n*_5_ = ((*V*^2^ - 1)*A*^2^ + 8*Eω* + 2*B*^2^), *n*_6_ = - (16*E*^2^*ω*^2^ + 8*EB*^2^*ω* + *B*^ 4^), *n*_7_ = ((*V*^2^ - 1)^2^*A*^4^ - 256*E*^2^*ω*^2^ - 128*B*^2^*Eω* -16*B*^4^), *ω* = *A* - *C*, *V*, *A*, *B*, *C*, *E* are free parameters.

For set 1, substituting Eq. (16) into Eq. (15), along with Eq. (7) and simplifying, yields following traveling wave solutions, if *C*_1_ = 0 but *C*_2_ ≠ 0; *C*_2_ = 0 but *C*_1_ ≠ 0 respectively:

Substituting Eq. (16) into Eq. (15), along with Eq. (8) and simplifying, our exact solutions become, if *C*_1_ = 0 but *C*_2_ ≠ 0; *C*_2_ = 0 but *C*_1_ ≠ 0 respectively:

Substituting Eq. (16) into Eq. (15), together with Eq. (9) and simplifying, our obtained solution becomes:

Substituting Eq. (16) into Eq. (15), along with Eq. (10) and simplifying, we obtain following traveling wave solutions, if *C*_1_ = 0 but *C*_2_ ≠ 0; *C*_2_ = 0 but *C*_1_ ≠ 0 respectively:

Substituting Eq. (16) into Eq. (15), together with Eq. (11) and simplifying, our obtained exact solutions become, if *C*_1_ = 0 but *C*_2_ ≠ 0; *C*_2_ = 0 but *C*_1_ ≠ 0 respectively:

where η = *x-Vt*.

Again for set 2, substituting Eq. (17) into Eq. (15), along with Eq. (7) and simplifying, our traveling wave solutions become, if *C*_1_ = 0 but *C*_2_ ≠ 0; *C*_2_ = 0 but *C*_1_ ≠ 0 respectively:

Substituting Eq. (17) into Eq. (15), along with Eq. (8) and simplifying yields exact solutions, if *C*_1_ = 0 but *C*_2_ ≠ 0; *C*_2_ = 0 but *C*_1_ ≠ 0 respectively:

Substituting Eq. (17) into Eq. (15), along with Eq. (9) and simplifying, our obtained solution becomes:

Substituting Eq. (17) into Eq. (15), together with Eq. (10) and simplifying, yields following traveling wave solutions, if *C*_1_ = 0 but *C*_2_ ≠ 0; *C*_2_ = 0 but *C*_1_ ≠ 0 respectively:

Substituting Eq. (17) into Eq. (15), along with Eq. (11) and simplifying, our exact solutions become, if *C*_1_ = 0 but *C*_2_ ≠ 0; *C*_2_ = 0 but *C*_1_ ≠ 0 respectively:

where η = *x-Vt.*

Similarly, for set 3, substituting Eq. (18) into Eq. (15), together with Eq. (7) and simplifying, yields following traveling wave solutions, if *C*_1_ = 0 but *C*_2_ ≠ 0; C_2_ = 0 but C_1_ ≠ 0 respectively:

Substituting Eq. (18) into Eq. (15), along with Eq. (8) and simplifying, we obtain following solutions, if *C*_1_ = 0 but *C*_2_ ≠ 0; *C*_2_ = 0 but *C*_1_ ≠ 0 respectively:

Substituting Eq. (18) into Eq. (15), along with Eq. (9) and simplifying, our obtained solution becomes:

Substituting Eq. (18) into Eq. (15), along with Eq. (10) and simplifying, yields following exact traveling wave solutions, if *C*_1_ = 0 but *C*_2_ ≠ 0; *C*_2_ = 0 but *C*_1_ ≠ 0 respectively:

Substituting Eq. (18) into Eq. (15), along with Eq. (11) and simplifying, our obtained exact solutions become, if *C*_1_ = 0 but *C*_2_ ≠ 0; *C*_2_ = 0 but *C*_1_ ≠ 0 respectively:

where *η* = *x-Vt*.

### Results and discussions

It is worth declaring that some of our obtained solutions are in good agreement with already published results which are presented in the following tables (Table 1).

**Table 1:** **Comparison between Neyrame et al.** (2010) **solutions and our solutions**

Neyrame et al. (2010) solutions	Obtained solutions
i. If *C*_1_ = 0 and *u*(*ξ*) = 4*v*_21_(*η*), Case 1 becomes:	i. If *A* = 1, *C* = 0, Ω = λ^2^-4*μ*, *B* = 1, *E* = 1, *V* = 1, then the solution is
ii. If *C*_1_ = 0 and *u*(*ξ*) = 4*v*_23_(*η*), Case 2 becomes:	ii. If *A* = 1, *C* = 0, Ω = λ^2^-4 *μ*, *B* = 1, *E* = 1, *V* = 1, then the solution is
iii. If *u*(*ξ*) = 4*v*_25_(*η*), Case 3 becomes:	iii. If *A* = 1, *C* = 0, *B* = 1, *E* = 1, *V* = 1, then the solution is

Beside this table, we obtain further new exact traveling wave solutions , , , ,  in this article, which have not been reported in the previous literature.

### Graphical representation of the solutions

**Figure 1:**
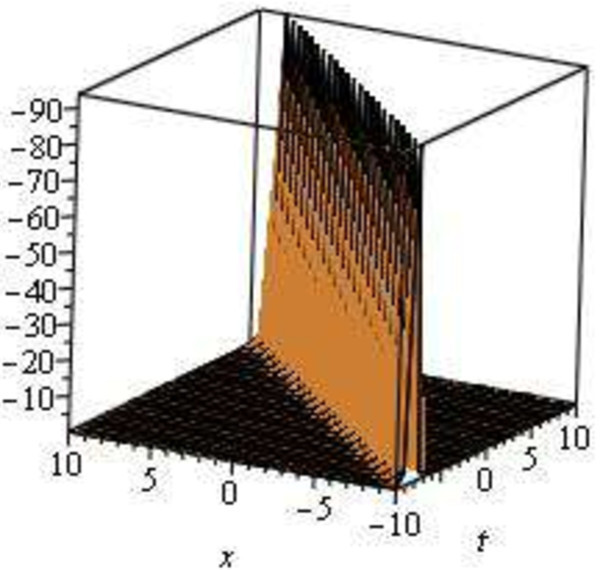
**Modulus plot singular soliton solution, shape of**  **when** ***A*** **= 4,** ***B*** **= 0,** ***C*** **= 1,** ***E*** **= 1,** ***V*** **= 1,** ***d*** **= 0 and -10 ≤** ***x*** **,** ***t*** **≤ 10.**

**Figure 2:**
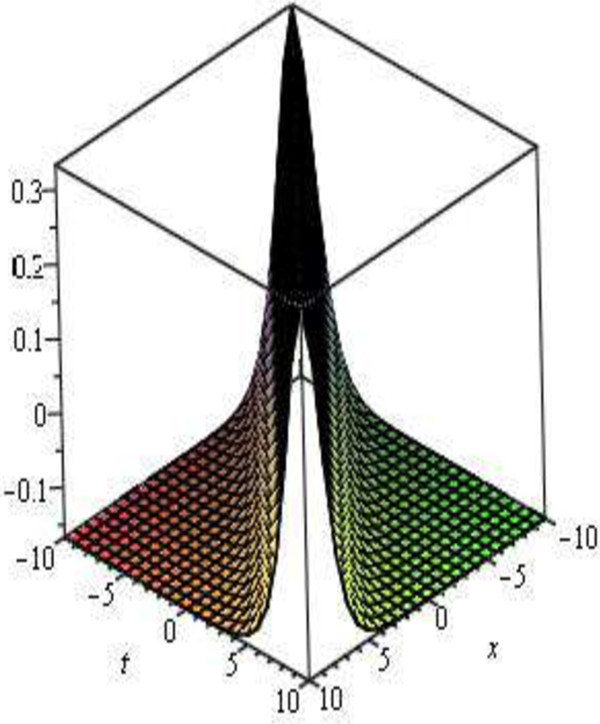
**Bell-shaph sec** ***h*** ^**2**^ **solitary traveling wave solution, shape of**  **when** ***A*** **= 2,** ***B*** **= 0,** ***C*** **= 1,** ***E*** **= 1,** ***V*** **= 1 and -10 ≤** ***x*** **,** ***t*** **≤ 10.**

**Figure 3:**
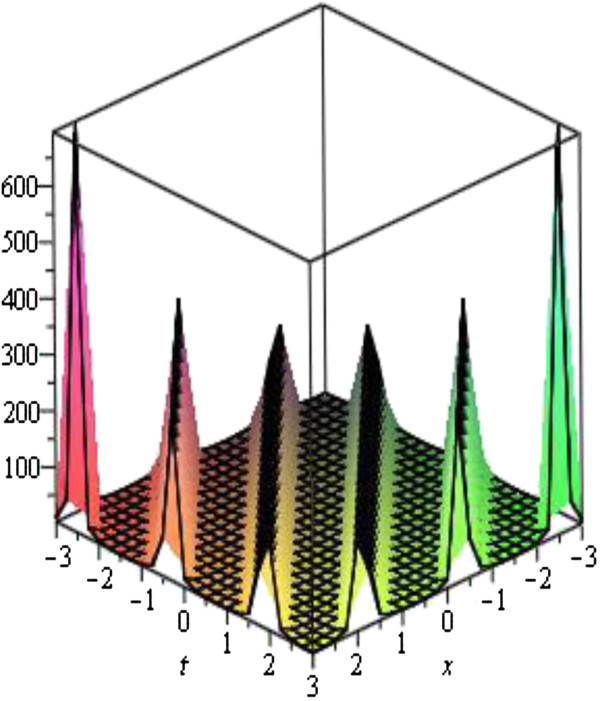
**Modulus plot of periodic wave solutions, shape of**  **when** ***A*** **= 1,** ***B*** **= 0,** ***C*** **= 2,** ***E*** **= 2,** ***V*** **= 1 and -10 ≤** ***x*** **,** ***t ≤ 10*** **.**

**Figure 4:**
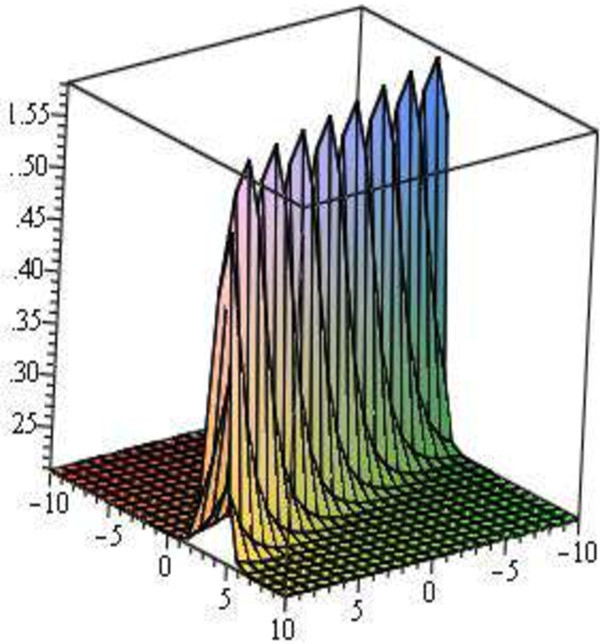
**Modulus plot of soliton wave solutions, shape of**  **when** ***A*** **= 4,** ***B*** **= 0,** ***C*** **= 1,** ***E*** **= 1,** ***V*** **= 3,** ***d*** **= 1 and -10 ≤** ***x*** **,** ***t*** **≤ 10.**

**Figure 5:**
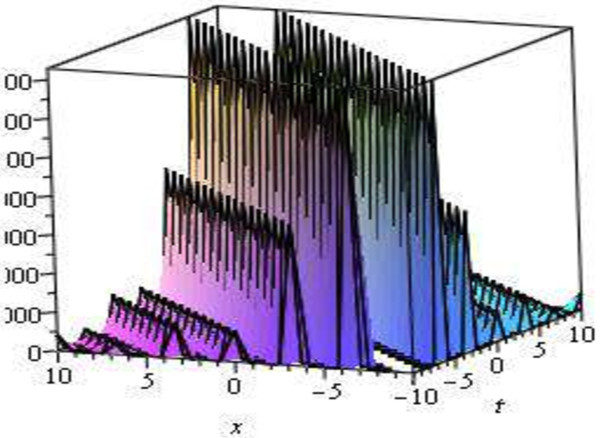
**Modulus plot of singular periodic wave solutions, shape of**  **when** ***A*** **= 1,** ***B*** **= 0,** ***C*** **= 2,** ***E*** **= 2,** ***V*** **= 1,** ***d*** **= 1 and -10 ≤** ***x*** **,** ***t*** **≤ 10.**

The graphical illustrations of the solutions are given below in the figures with the aid of Maple (Figures 1, 2, 3, 4 and 5).

The solutions corresponding to , ,  is identical to the solution , the solution corresponding to  is identical to the solution , the solution corresponding to  is identical to the solution , the solution corresponding to ,  is identical to the solution  and the solution corresponding to ,  is identical to the solution .

## Conclusion

In this paper, we obtain the traveling wave solutions of the Boussinesq equation by using the new approach of generalized (*G*'*/G*) -expansion method. We apply the new approach of generalized (*G*'*/G*)-expansion method for the exact solution of this equation and constructed some new solutions which are not found in the previous literature. This study shows that the new generalized (*G*'*/G*)-expansion method is quite efficient and practically well suited to be used in finding exact solutions of NLEEs. Also, we observe that the new generalized (*G*'*/G*)-expansion method is straightforward and can be applied to many other nonlinear evolution equations.
